# Language Matters: Development of an Objective Structured Language Test for Foreign Physicians – Results of a Pilot Study in Germany

**DOI:** 10.3205/zma001210

**Published:** 2019-02-15

**Authors:** Holger Lenz, Ansgar Opitz, Dana Huber, Fabian Jacobs, Wolfgang Gang Paik, Jörg Roche, Martin R. Fischer

**Affiliations:** 1 Klinikum der Universität München, Institut für Didaktik und Ausbildungsforschung in der Medizin, München, Germany; 2 LMU München, Lehrstuhl für Empirische Pädagogik und Pädagogische Psychologie, München, Germany; 3 LMU München, (ehem.) Institut für Deutsch als Fremdsprache, München, Germany; 4 LMU München, Medizinstudierender, München, Germany; 5 LMU München, Institut für Deutsch als Fremdsprache, München, Germany

**Keywords:** medical language, exam, foreign physicians

## Abstract

**Objective: **To develop a scientifically sound and standardized medical language examination for the State of Bavaria according to the requirements set forth by the 87^th^ Conference of State Health Ministers. This *Sprachtest für Ausländische Mediziner* (SAM, Language Test for Foreign Physicians) ought to become part of the licensing procedure for foreign physicians in Germany. Using testing stations that are situation-based, it will assess medical language competence and communication skills at the proficiency level of C1.

**Methods: **Case scenarios for four mini-interviews of 10 minutes each were developed. For the written part of the exam, consisting of two separate testing stations with a combined duration of 40 minutes, one video of a physician taking a patient’s history and one annotated set of laboratory results were developed. Based on the analysis of existing scientific literature as well as real-life examples, features and characteristics of professional medical language were identified. This served as the basis for the development of itemized rating scales for each of the testing stations. The exam was validated in three simulated trial runs. Each run was video-recorded and subsequently graded by a team of test-raters.

**Results: **19 participants took part in the three trial runs. A benchmark (gold standard) could be set for 18 of these. A ROC-analysis yielded an AUC-value of .83. This confirmed the predictive quality of the SAM-test. The reliability of the SAM-test could be calculated for only ten participants. The internal consistency, calculated with the use of Cronbach’s Alpha, was .85. The pass/fail mark was calculated based on the Youden-Index and yielded a result of >60%.

**Conclusion:** The SAM-test presents a statistically valid medical language examination with a high level of objectivity. As required, it tests language proficiency at the level of C1 and uses authentic communication scenarios within a standardized test setting. Additional studies with larger test samples will help to further validate this test and thus guarantee a higher degree of reliability.

## Zusammenfassung

**Zielsetzung: **Entwicklung einer wissenschaftlich fundierten und standardisierten Fachsprachenprüfung für das Bundesland Bayern gemäß den Vorgaben der 87. Gesundheitsministerkonferenz (GMK). Der SAM – Sprachtest für ausländische Mediziner soll Teil des Approbationsverfahrens ausländischer Ärzte und Ärztinnen sein. In situativen Prüfungsstationen soll er fachsprachliche und kommunikative Kompetenzen auf C1-Niveau abprüfen.

**Methodik: **Für vier je zehnminütige Mini-Interviews wurden Fallvignetten ausgearbeitet, für die 40-minütige schriftliche Prüfungsstation, die aus zwei Teilaufgaben besteht, wurde ein Video einer Anamnese sowie kommentierte Laborergebnisse als Basis der Aufgabenstellungen erstellt. Fachsprachlichen Kompetenzen wurden anhand von Analysen wissenschaftlicher Literatur und empirischer Beispiele fixiert und als Items zu Bewertungsskalen für jede Teilstation zusammengefasst. In drei Simulationen wurden die Prüfungen per Video (SAM-Prüfungssoftware) aufgezeichnet und im Anschluss von Bewerterteams bewertet.

**Ergebnisse: **19 Probanden nahmen an drei Simulationen teil. Eine Goldstandardsetzung konnte bei 18 von ihnen durchgeführt werden. Eine ROC-Analyse ergab einen AUC-Wert von .83, was die prognostische Qualität des SAM bestätigt. Die Reliabilität des SAM konnte nur für zehn Probanden berechnet werden. Die mit Cronbachs Alpha berechnete interne Konsistenz betrug .85. Die Bestehensgrenze wurde mithilfe des Youden-Index ermittelt. Für den SAM ergab sich dabei die Grenze von >60%.

**Schlussfolgerung: **Mit dem SAM wurde eine valide Fachsprachenprüfung mit hoher Test-Objektivität vorgelegt, die in authentischen Kommunikationssituationen und einem standardisierten Setting die Fachsprachenkenntnisse im geforderten C1-Niveau abprüft. Mit weiteren Erprobungen und einer größeren Stichprobe kann der SAM weiter validiert und eine höhere Test-Reliabilität sichergestellt werden.

## 1. Introduction

“Anyone who focuses only on the slightly increasing number of physicians closes his eyes to the whole truth. In reality, the gap between demands for medical care and the capacities to give it continues to widen steadily.” [1]. Thus commented the president of the German Medical Association, Frank Ulrich Montgomery, on the nationwide statistic for physicians from 2016. For some time now, buzzwords such as “shortage of physicians” and “shortage of skilled workers” have been circulating through the public discourse on health policy [2]. More and more physicians from foreign countries continue to close the gap: within the past five years, their number in Germany has nearly doubled. It reached a record high in 2016 with a total of 41.658 [3].

As they go through the process of integrating into their everyday professional lives, however, foreign physicians are confronted with a number of technical, administrative and cultural challenges that are partly driven by a lack of language proficiency. Insufficient or deficient communication skills often result in a lower quality of treatment, lower levels of patient satisfaction as well as intercollegiate conflicts and therefore pose a significant threat to patient safety. In extreme cases, failure to communicate successfully can be the decisive factor whether a patient dies or lives [4], [5], [6], [7], [8]. Proficient communication that clears misunderstandings and prevents them is a vital element of medical practice [9]. 

**Figure 1:**
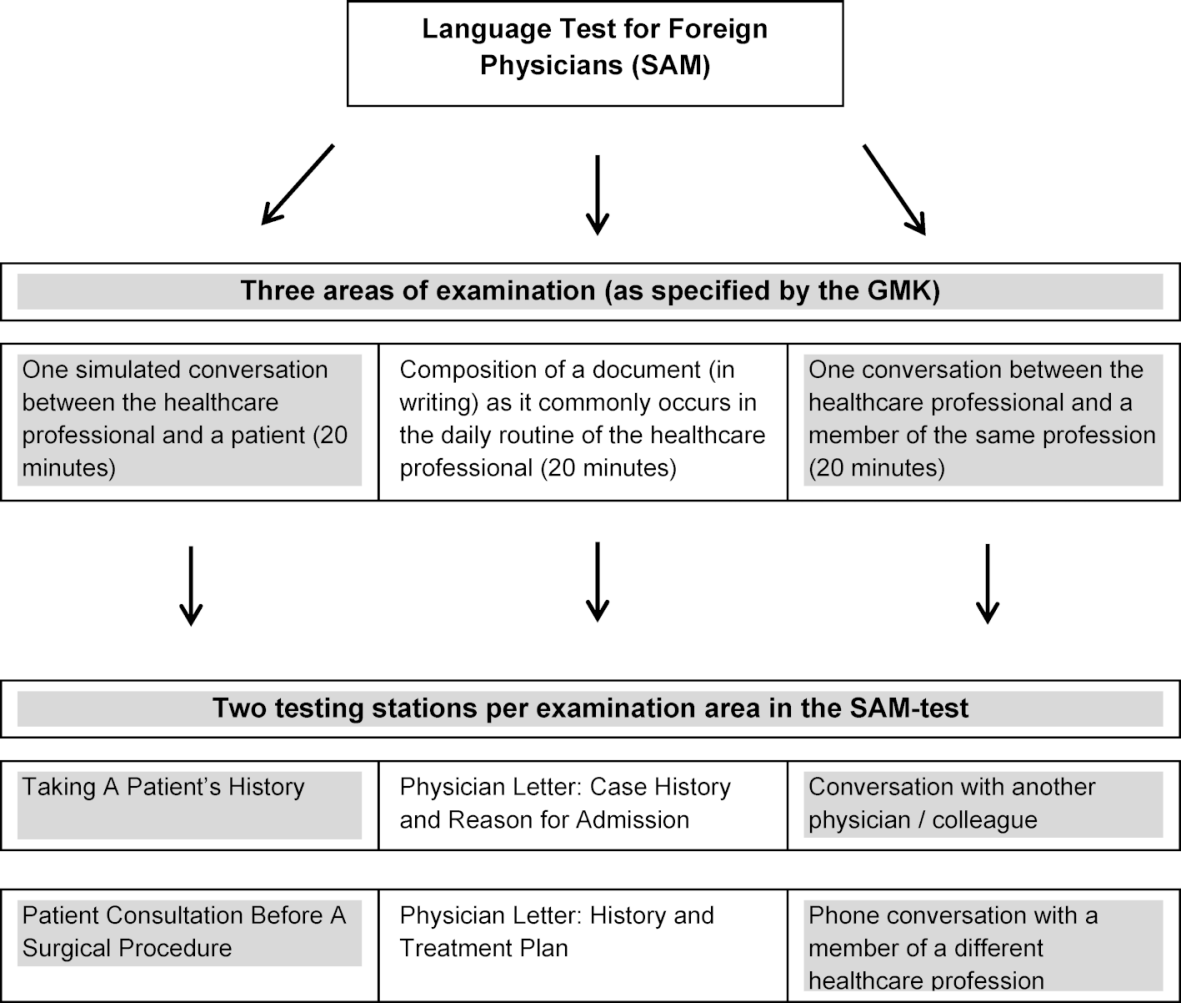
Schematic design of the language test for foreign physicians

Therefore, the 87^th^ *Gesundheitsministerkonferenz* (GMK, Conference of State Health Ministers) resolved to make obligatory a nationwide language exam for healthcare professionals and, at the same time, specified a number of minimum requirements. Those requirements include one simulated conversation between the healthcare professional and a patient; the composition of a document in written form as it commonly occurs in the daily routine of the healthcare professional; and a conversation with a member of the same profession. Each part should last 20 minutes [10] (cp. Abbildung 1 (Fig. 1)). To this date, there are no common standards for the theoretical and methodological framework of this test. The formal requirements as established by the GMK refer mostly to the language level C1 when used as an expression of language in a professional context or setting. 

It is true that the requirements did create the necessary framework for higher standards of language proficiency. The responsibility to guarantee language exams of high quality, however, was shifted to the individual states. As can be seen from an overview issued by the *Marburger Bund*, the conceptualization of the actual exam varies greatly from state to state [11]. The lack of a common national exam, however, causes the risk of so called “exam tourism”. This means that foreign medical professionals will try to pass the examination in those states, in which the exam is supposedly easier to pass than in other states. In 2016, the state of Bavaria represented by the *Staatsministerium für Gesundheit und Pflege* (StMGP, State Healthcare Department), commissioned an interdisciplinary research team from the medical faculty, the Institute for Medical Education, the Department of German as a Foreign Language and from psychometrics at the *Ludwig-Maximilians-University* (LMU) with the development of a valid, reliable, fair, authentic, objective and viable *Sprachprüfung für ausländische Mediziner* (SAM, Language Test for Foreign Physicians). 

In the Anglo-American world, the Australian test model can be considered as the leading model, since it, too, is based on similar scientific and methodological standards [12]. An analysis of this model, however, has shown that international test models can be used only as a general guideline. Even the Australian model does not meet all scientific criteria of test development [13], [14]. This made it absolutely necessary to create an independent methodological foundation for the SAM-test. This article outlines the design of the SAM-test and discusses the initial testing phase along with its results.

## 2. Project Description and Methodology

Considering the requirements as stated in the resolution of the 87th GMK, the research team developed a design concept that primarily focused on meeting the quality criteria of objectivity, reliability, validity and authenticity [10], [15], [16], [17].

### 2.1. Design of the examination

The schematic design of the exam can be seen as displayed in figure 1 (Fig. 1). *Taking A Patient’s History and Patient Consultation Before A Surgical Procedure* were chosen as topics for the part of doctor-patient-communication. When *Taking A Patient’s History*, the examination candidate has to verbally obtain information relevant to the patient’s medical condition, allow adequate time for the patient to report about his symptoms and create an atmosphere of respect. At the same time, the candidate’s ability to understand spoken language is tested. During the *Patient Consultation*, the focus shifts towards the transmission of information. The physician needs to explain the process of the upcoming surgical procedure, point out potential risks and give detail instructions about post-surgical precautions and measures. Focus of this test section is the use of vernacular (avoiding technical medical terms), ascertaining that the patient has understood all relevant information as well as verbally and nonverbally expressing empathy toward the patient’s questions and concerns.

A prototypical communication situation for the part ‘intercollegiate communication’ is the presentation of a patient’s case to others: *Relating A Patient’s History* and condition to the senior physician. In this section of the test, which is also based on a simulated case scenario, the examinee has to use technical medical language and terminology to demonstrate successful communication with a colleague (here: a senior physician). Both the act of relaying information as well as stating clear and clarifying questions should be done in a concise way and with accuracy. 

In contrast with other medical language exams in Germany and as required by the GMK, the SAM-test also includes the examination of communicative proficiency when it comes to conversations between physicians and professionals from other healthcare professions [10]. The* Instructive Conversation With A Nurse* was thus chosen as a typical scenario for this type of communication format. In this test section, clearly stated instructions have to be given to a nurse. This should also happen while using appropriate technical language and terminology in a respectful atmosphere. 

For the written part of the exam, an analysis of 200 physician letters from the fields of surgery and internal medicine at the university hospital of the LMU revealed that physician letters generally consist of four structural elements. Two of those, *Case History and Reason for Admission* and *History and Treatment Plan* were included in the SAM-test because of their high level of difficulty. The written part tests the examinee’s ability to receive and process language input along with the ability of making verbal expression in written form. 

Cases from the subject areas of general medicine, internal medicine and surgery were chosen for the case scenarios. These areas generally correspond with the content areas of a subsequent examination that foreign physicians from countries which are not members of the European Union have to take in order to demonstrate their medical-technical know-how at the level of the 3^rd^ State Examination before receiving their license to practice. Thus, setting the focus on these subject areas can be seen as justified regardless of the personal specialty area of each candidate.

Case scenarios were kept as general as possible in order to avoid focusing the exam too much on content specific to one area of medical practice. During the *Patient Consultation*, for example, examinees deal with scenarios from common surgical procedures such as a thyroidectomy or a tonsillectomy.

#### 2.2. Format of the examination

The OSCE-format (Objective Structured Clinical Examination) was chosen for the SAM-test to meet the real (authentic) demands of everyday professional practice and at the same time create conditions comparable to those existing for medical students, who have to prove their medical know-how at a university. According to Miller, OSCE-exams offer the opportunity to not simply reproduce knowledge, but to show what one has learned in a practical, context-driven setting [17]. From the viewpoint of medical educators, OSCEs have established themselves as reliable and valid instruments when it comes to testing clinical-practical knowledge [18]. Brandes and Bagnasce et al. have further shown that OSCEs are well suited as a methodological setting for measuring communication skill levels in cultural and professional contexts [19], [20]. Analogous to the OSCE-concept of multiple short test scenarios with a length of five to ten minutes, the SAM has been designed with two testing stations of ten minutes each for each one of the two areas that examine oral proficiency (cp. figure 1 (Fig. 1)). This leads to an increase in reliability, since the performance of the examinee can be observed four times in four different contexts. Additionally, ten minute scenarios more realistically represent the time frame available to physicians during their daily routine, which therefore increases the level of authenticity of the test. 

#### 2.3. The problem of interdependent testing station results

Current medical language examinations often use one case scenario throughout the entire test. From a psychometric point of view, however, this concept presents challenges: if *one* case scenario is used throughout the entire exam, this creates a dependency between the assessment items for each testing station of the exam: the results in one area no longer depend solely on the performance in that area, but also on the performance in preceding test areas [15].

Additionally, the “one case scenario” model leads to a drastic reduction of* fairness*: if the candidate is accidentally tested in an area that s/he is especially familiar with due to former experience or past medical education, his or her test performance is automatically better. Finally, using a model in which test areas are independent of each other alleviates the exchange of case scenarios that need to be removed from the exam due to repeated use: if a test consist of multiple case scenarios, it is possible to compare the level of difficulty of a new case scenario with the level of difficulty of existing ones; if, however, a test consists of only one case scenario, the exchange of that one scenario automatically leads to the exchange of the entire test. This, however, makes it impossible to assess the level of difficulty of the new case scenario in relation to other case scenarios. Therefore, different case scenarios with multiple testing stations have been used for the SAM-test.

#### 2.4. Implementation and assessment

Every language examination that aims at testing the examinee's productive and receptive language abilities has to create communication situations that are as realistic as possible (authentic) and as reproducible as possible (objective and fair). This ensures that all candidates are tested within the same communicative contexts. To create such standardized communication settings, the SAM-test makes use of trained actors for the roles of the “patient” and the “nurse”. The role of the senior physician is filled by an actual, real-life physician. 

Both the actor simulating the patient and the real-life physician attend multiple training units to prepare for their roles. The main emphasis of the training units is to create standardized test settings (objectivity, fairness) and to evoke language patterns that are specific to each case scenario. A script for the simulated patient with detailed instructions and additional questions was developed. 

Current medical language examinations in other German states assess the examinee's performance in a synchronous way: a group of raters present in the testing room observe the candidate's performance and evaluate it, often with the help of standardized assessment sheets. Synchronous assessments of oral performance, however, are problematic in many ways: what is expressed verbally is fleeting by nature and cannot be reviewed; assessment is also made “out of the (ongoing) situation” and raters are often participants in the communication situation. 

Asynchronous assessment with raters who are not part of the communication situation and who only assess the oral parts of the exam, however, allows for repeated, independent and standardized listening to the candidate’s performance and thus increases the objectivity of results. Therefore, oral test parts are video-recorded in the SAM-test. This method of testing and performance evaluation, called VOSCE (Video-Recorded Objective Structured Clinical Examination), has successfully proven to be a feasible, reliable and valid method to assess communication ability in other medical contexts [21], [22], [23]. Since storing and accessing recorded test data can be problematic in view of strict data protection and privacy laws, a special software program was developed. This program records each performance through an external camera attached to a laptop computer and stores the recorded, pseudonymized data on a password-protected server, which allows for secure access of recordings by the team of test raters at a later point in time. This team consists of one physician and one linguist with a background in German as a Second Language theory and test methodology. An itemized rating scale was developed for each testing station (*History Taking, Patient Consultation*, etc.). For each item, the rater must choose between three different possibilities: “Standard was met”, “Standard was not met” and “Not sure”. The option “Standard was met” is equivalent to one point, the option “Standard was not met” to 0 points and the option “Not sure” to 0.5 points. All items are categorized according to the typical structure of the professional language in use, the linguistic pattern or style, the behavior in the communication situation as well as the global impression of the performance in the communication situation as a whole. Each rating scale consists of between 11 and 17 items, which adds up to a total of 83 items for the SAM-test (cp. table 1 (Tab. 1)). An example of a rating scale for the subpart *History Taking* can be found in attachment 1 .

A supplementary sheet for each rating scale explains the intended use of the items and gives case-specific examples. This complies with the requirements of the Association of Language Testers in Europe (ALTE) for language test assessment procedures [24], and increases the probability of a standardized and consistent rating process. Additionally, the test-developers provided a training session (ca. one hour) for each new team of test-raters in order to explain the rating process and answer any pending questions. 

Rating of test performances first occurs individually. Afterwards, the team of raters has to agree unanimously whether a candidate passes or fails the test. After assessing the test performance individually, raters compare their results and must reach a consensus for any diverging assessment of the rating scale items. The cumulative result of all six testing stations finally decides whether a candidate passes the test or not. 

## 3. Pilot Testing of the SAM-Test

### 3.1. Implementation

During the pilot testing phase, the SAM-test was validated in three simulated trial runs. A total of 19 candidates participated in the trial runs. These came either from the pool of international medical students at the LMU (n=10) or from the pool of international physicians who live in Germany, but do not yet have their license to practice medicine (n=9). With the help of these simulations, it could be determined how feasible it was to implement the design of the SAM-test. Additionally, the results were used to determine to which degree rater evaluations of performances are in agreement, to measure reliability, to evaluate the prognostic ability of the test and to determine the pass/fail mark. In order to determine the pass/fail mark and to understand the prognostic ability of the test, a benchmark (gold standard) was used: In addition to the (regular) assessment of participants’ performance in the SAM-test by a team of test raters, an expert team consisting of two professionals from the subject areas “Medicine” and “German as a Foreign Language” with many years of experience in assessing communication performances joined the rating process. These experts used a global rating system to determine whether candidates had reached the minimal requirement of the C1 language level. Comparing the itemized results of the regular rating team with the assessment of the two expert raters (which was used as the gold standard) allowed for evaluating the quality of the SAM-test as well as for setting the pass/fail mark. 

#### 3.2. Results

It is best to use Cohens Kappa to determine as to which degree the two raters' performance evaluations are in agreement. This indicates to what extent the consensus of the two raters is higher when compared with a set of randomly generated evaluations. Possible values range from 0 to 1. Through the use of training sessions, the SAM-team was able to raise the consensual value of evaluations from .49 to .72. At the end of the pilot testing phase, the percentage of consensual evaluations was at 88% (cp. to 80% at the outset). 

Because of missing data as well as minor adjustments of the rating scales between the first and subsequent trial runs, the reliability of the overall SAM-scale could only be calculated for ten candidates and 81 items. The internal consistency of this set of 81 items, calculated with the use of Cronbach's alpha, was .85. The reliability values for each testing station (for which there is more data) can be seen in table 1 (Tab. 1). 

On average, all candidates fulfilled M=55% (SD=22%) of the 83 items of the six rating scales. A benchmark (gold standard) could be set for 18 candidates. Five were rated as reaching the minimum qualification of the C1 language level. The performance result of M=69% (SD=19%) of these five candidates was higher than the performance result of those who did not achieve the minimum requirements according to the benchmark (gold standard) (M=46%, SD=14%). To accurately examine the prognostic ability of the SAM-test (in relation to the eligibility of the candidates), a ROC-analysis was used (receiver operating characteristics) [25]. This analysis determines to what extent the performance in a test corresponds with the “actual” proficiency of the candidate (represented by the benchmark). The global quality level of the test can thus be quantified by using the AUC-value (area under the curve). The AUC-value can range from 0 to 1. An AUC-value of 0.5 means that the test is no better than mere chance in determining which candidate is qualified and which one is not. An AUC-value of 1 means that the assessment of all candidates is correct. For the SAM-test, an AUC-value of .83 was determined. According to current test methods, this shows a strong effect and emphasizes the prognostic quality of the SAM-test [26].

Moreover, the pass/fail mark was determined with the help of the ROC-analysis. To do this, the Youden-Index was used [27]. This index combines the sensitivity (the number of candidates who are qualified and are correctly identified as such by the test) and specificity (the number of candidates who are not qualified and are correctly identified as such by the test) of the test into one single value. Higher values are desired. A pass/fail mark of 50% produced a value of .49. At this mark, the values of sensitivity and specificity were at .80 and .69 respectively. The PPV (positive predictive value; the probability that a candidate is truly qualified once the pass/fail mark has been reached) lies at .50 for this threshold, and the NPV (negative predictive value; the probability that a candidate is truly unqualified if the pass/fail mark is not reached) at .90. A pass/fail mark of >60% results in a Youden-Index value of .52. Even though the sensitivity drops to .60, the specificity value rises to .92. The PPV is .75 and the NPV .86. If the Youden-Index is used as a criterion and one assumes that the highest priority of any medical language examination is to prevent possible damage to the general public, a more conservative threshold of >60% should be used. In this context, “conservative” means that a candidate whose performance falls in the borderline area between qualified and unqualified is deemed as unqualified. The data of the trial runs even allow for the possibility to raise the pass/fail mark to 70%. Without loss of sensitivity, this would result in a rise of the specificity value to 1. However, since the distribution of data suggests that at such a pass/fail rate, the sensitivity value would drop off once larger data sets are used, and since the specificity value at the >60% mark is already very high (.92%), a pass/fail mark of >60% is suggested for the SAM-test. Table 2 (Tab. 2) represents an overview of the most important statistical results.

## 4. Discussion and Conclusion

Good results were achieved especially in the areas of fairness, authenticity and objectivity. In this context, it is important to again emphasize the importance of coaching all actors who participate as simulated patients in the communication situation. Only if the simulated patient acts in a consistent matter towards each and every candidate can a reproducible test environment be guaranteed. The resulting increase in test-objectivity in turn has a positive effect on the reliability and validity of the test. Inversely, the low reliability value of the testing station *Patient Consultation* could possibly be explained by referring to the occasional but unintended observation of simulated patients giving assistance during the communication situation. It is possible that simulated patients (who do not have a background in medicine) give cues to weaker candidates out of a feeling of empathy. This would reduce the systematic variance of results and thus affect reliability. This and other data collected within the context of this project about the respective peculiarities and challenges that both simulated patients and exam candidates encounter within each communication situation of the test can therefore serve as an initial basis for the development of a standardized, scientifically verified training method. 

Another strength of the SAM-test lies within the concept of evaluating test performances in an asynchronous manner. Test raters who experience the communication situation “live” or are even part of the communication situation themselves increase the risk of introducing *bias* into the rating of the candidate's performance. The model of asynchronous assessment of test performance used in the SAM-test contributes to a fair and objective evaluation of all examinees und thus reduces the risk of legal complaints on the part of exam candidates. 

The validity values of the SAM-test based on the ROC-analysis of data from the pilot testing phase are promising. This is especially so considering that, according to the benchmark (gold standard), the rate of qualified candidates was low, which in turn complicates the process of identifying qualified candidates. When analyzing the results, it is furthermore important to bear in mind that half of the participants in the trial runs were foreign students. Since students have less experience and knowledge than experienced physicians, it is possible that this contributed to a distortion of the collective performance results of all candidates. Within the sample group of experienced physicians, the rate of qualified candidates should thus be higher. It is further necessary to take into consideration that the relatively small sample group from all three trial runs implies a high level of uncertainty of all test parameters. A more systematic validation of the test is therefore absolutely necessary. For example, the overall good validity of rating scales during the pilot testing phase and subsequent performance assessment is at odds with the unsatisfactory reliability values of the rating scales for two testing stations (*Patient Consultation* and* Instructing a Nurse*). Future trials that intend to reduce the deficiency of above mentioned scales and aim at increasing the psychometric quality of all scales could therefore especially benefit from trial samples of larger size and consisting of a more homogenous group of candidates respective their language ability and proficiency. A more precise measurement of the reliability value of the SAM-test would thus be a natural consequence of a larger sample size. 

Another weakness of the SAM-test lies in the initial investment costs needed for setting up the test environment (software program and training of the simulated patients and raters). The longer the SAM-test runs, however, the more should its strengths serve to offset this disadvantage. 

Further action is needed regarding the distribution of the number of items for the rating scales. The item number for the scales of each individual testing station varies between 11 and 17. In order to give equal weight to the scale of each testing station, a retroactive adjustment is recommended to avoid the need to artificially increase or decrease the number of items. Before calculating the total sum value for the entire test, the point value achieved in each of the six testing stations would have to be multiplied by different coefficients so that the candidate can achieve exactly ^1^⁄_6_ of the total maximum points in each testing station. 

## 5. Outlook

To this date, the SAM-test represents the first and only scientific concept of a medical language test within Germany. In addition to the parameters set out by the GMK, quality standards of test and measurement theory such as objectivity, reliability, validity, authenticity, fairness and feasibility were closely adhered to as guiding principles of design and implementation. The SAM-test is also currently the only medical language examination in Germany that includes the aspect of inter-professional communication. In addition to the introduction of the communication setting between a physician and a nurse, it is conceivable to include further situations that produce inter-professional communication situations. In view of the goal to create and maintain a scientific and robust examination, it must be noted that further simulated trials are necessary.

It is further recommended to compare the SAM-test with other examinations to see how they measure up to the quality standards of test and measurement theory. Only then can the goal of a unified national exam, which reliably tests foreign physicians at the language level of C1 and thus guarantees patient safety, finally be reached. At the time of this writing, one additional comparative study with the goal of validating examinations currently used in the state of Bavaria is being planned. It is the professed aim of the test developers to see the SAM-test being used in the foreseeable future and thus to contribute to the lasting improvement of current methods of testing – not only in the State of Bavaria.

## Acknowledgements

For the sustainable support of the project, we would also like to thank Prof. Dr. med. Matthias Siebeck, Department of General, Visceral, Transplantation, Vascular and Thoracic Surgery of LMU Munich

## Funding

We would like to thank the Bavarian State Ministry for Health and Care (StMGP) for the support of the project under grant number G32g-G8517.1-2015/5-91.

## Competing interests

The authors declare that they have no competing interests. 

## Supplementary Material

Language Test for Foreign Physicians

## Figures and Tables

**Table 1:**
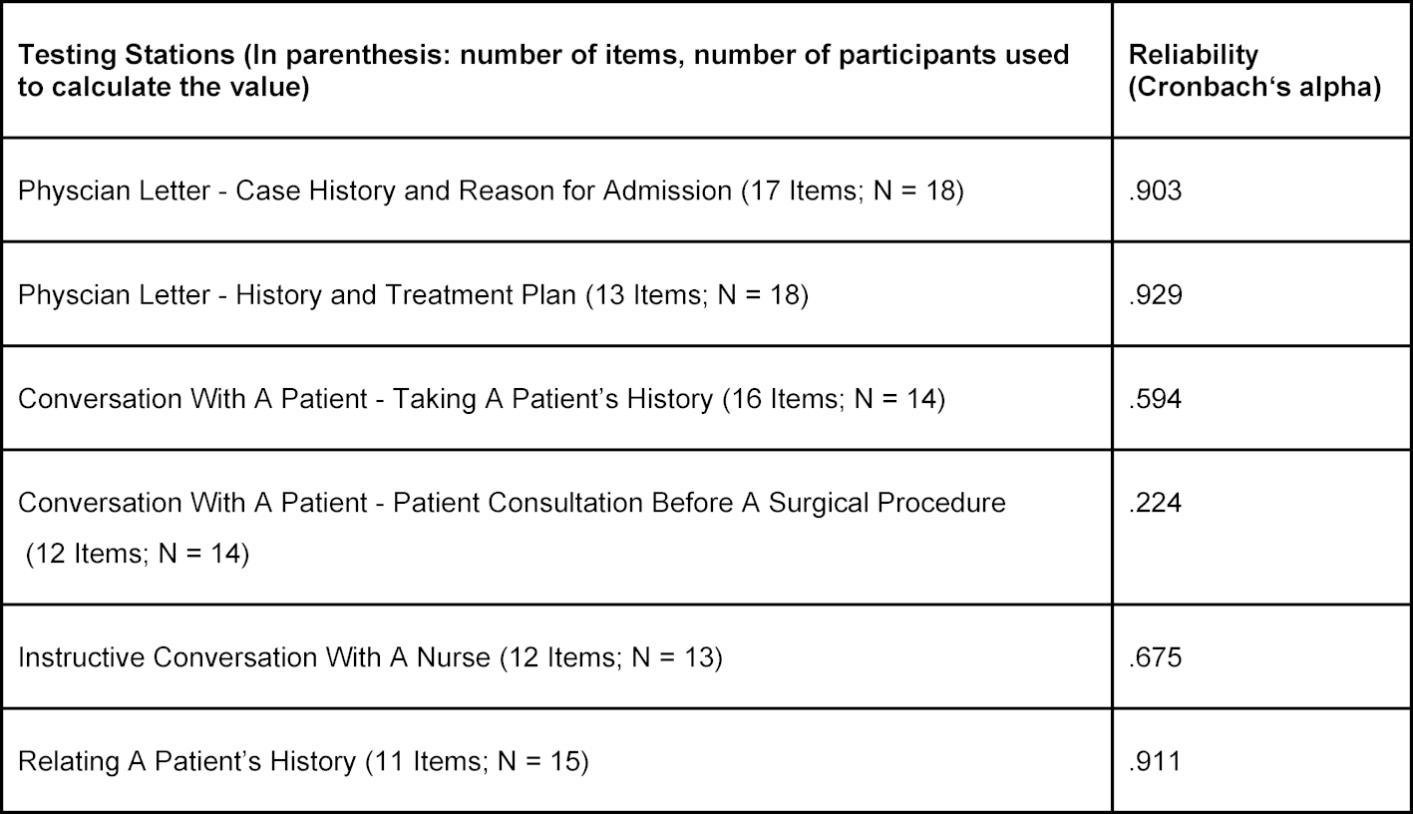
Reliability values of the individual testing stations of the SAM-test

**Table 2:**
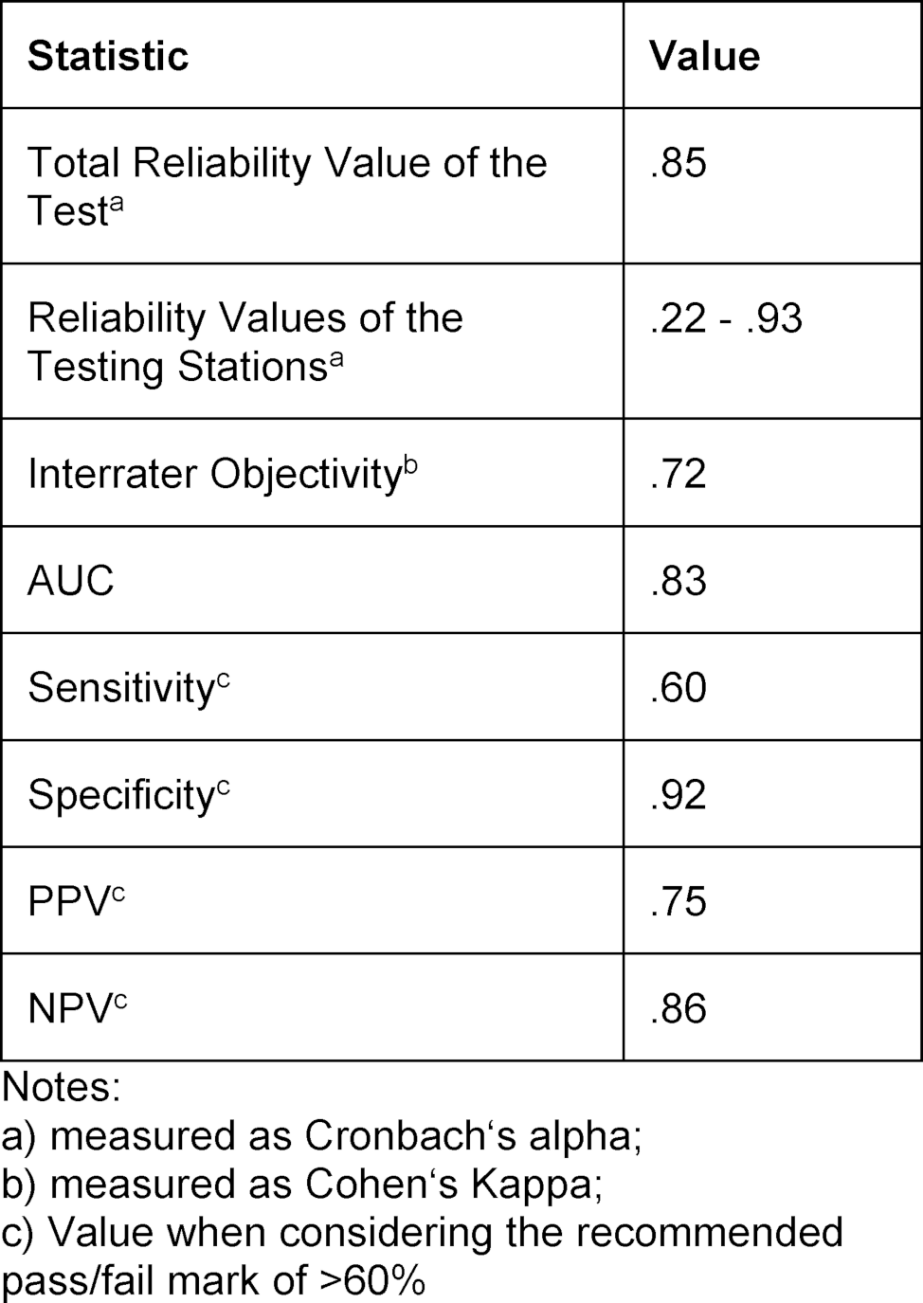
Overview of the most important statistical data of the SAM-test
