# Supplementary material for: Language Matters: Development of an Objective Structured Language Test for Foreign Physicians – Results of a Pilot Study in Germany
Source: GMS J Med Educ. 2019 Feb 15;36(1):Doc2. doi: 10.3205/zma001210 (PMC6390091; doi:10.3205/zma001210)
Supplement: Language Test for Foreign Physicians [file JME-36-2-s-001.pdf]

LMU | KLINIKUM DER UNIVERSITÄT MÜNCHEN

Language Test for Foreign Physicians

Rating Scale 'Physician-Patient-Communication' – Taking a Patient's History (Page 1/2)

Code: ____________________

Nr.	Item	Standard was met	Not sure	Standard was not met
	Ascertaining the medical condition			
1.1	The physician confirms the identity of the patient.	☐	☐	☐
1.2	The physician asks the patient to describe his condition.	☐	☐	☐
1.3	The physician enquires about the character of the condition in a precise way.	☐	☐	☐
1.4	The physician enquires about the progressive development of the condition (history of the present illness).	☐	☐	☐
1.5	The physician asks about the (current or regular) intake of medication by the patient.	☐	☐	☐
1.6	The physician asks about any pre-existing conditions.	☐	☐	☐
1.7	The physician invites the patient to speak about his health-related lifestyle.	☐	☐	☐
1.8	The physician inquiries about the patient's family diseases and social history.	☐	☐	☐
1.9	At the end of the conversation, the physician explains the next steps.	☐	☐	☐

Please continue the rating process on the next page!

Rating Scale 'Physician-Patient-Communication' – Taking a Patient's History (Page 2/2)

Nr.	Item	Standard was met	Not sure	Standard was not met
	Communicative Behavior			
2.1	The physician greets the patient in a polite manner.			
2.2	The physician addresses the patient in a respectful way.			
2.3	The physician treats the patient as a mature and competent conversation partner.			
2.4	The physician uses expressions that are in a common vernacular understandable to the patient (at the latest once the patient asks him to do so).			
2.5	The physician gives verbal or non-verbal feedback cues.			
2.6	The physician ensures (through paraphrasing or summarizing) that he has understood the information given by the patient correctly.			
	Overall Assessment			
3.1	There is the overall impression that the medical condition of the patient was captured sufficiently.			
3.2	All in all, the communicative behavior of the physician was appropriate and clear.			

Language Test for Foreign Physicians

Supplementary Sheet for the Rating Scale ‘Physician-Patient-Communication’ – Taking a Patient’s History (Part 1)

Case: Mr. Lauten / Tired and fatigued

Nr.	Item	Supplementary Information
1.1	The physician confirms the identity of the patient.	Reassure: direct question; indirect question; interrogative particles that secure information<br>Case: Markus Lauten<br>*Ex.: You are Mr. Lauten, correct?*
1.2	The physician asks the patient to describe his condition.	Prompt: direct prompt; open question;<br>Use of speech that invites narrative expression erzählraumöffnende Redemittel;<br>Subjective description: no use of leading question;<br>*Ex.: What made you come to us today? / How have you been? / Please tell me…*
1.3	The physician enquires about the character of the condition in a precise way.	Inquire: direct question; prompt; open or closed questions possible;<br>Accuracy: names [qualities] explicitly<br>*Ex.: Can you describe the symptoms? / Is it a shooting pain or rather a throbbing type of pain? / Is the pain throbbing or rather a shooting type of pain?*
1.4	The physician enquires about the progressive development of the condition (history of the present illness).	Inquire: open or closed questions regarding the [occurrence] [duration] [frequency] of the symptoms; regarding the [change] of symptoms / pain since they first occurred<br>*Ex.: When did you first notice the sore throat? Has the pain increased since then?*
1.5	The physician asks about the (current or regular) intake of medication by the patient.	Inquire: open and closed questions<br>*Ex.: Are you taking any medications? … Which? / Which medications are you currently taking?*
1.6	The physician asks about any pre-existing conditions.	Inquire: closed questions<br>*Ex.: Have you had these or similar symptoms before?*
1.7	The physician invites the patient to speak about his health-related lifestyle.	Prompt: direct prompt; closed questions<br>Questions about the use of tobacco, alcohol, etc.<br>*Ex.: Do consume alcohol on a regular basis? Do you use tobacco?*
1.8	The physician inquiries about the patient’s family diseases and social history.	Inquire: open or closed questions; gives examples of hereditary [diseases]<br>The physician inquires about the patient’s profession or relevant medical conditions in the patient’s family history<br>*Ex.: What do you do for work? / Have there been cases of … in your family?*
1.9	At the end, the physician explains the next steps.	*Ex.: Do you have any further questions? … Now I would like to proceed with the physical examination*

Language Test for Foreign Physicians

Supplementary Sheet for the Rating Scale 'Physician-Patient-Communication' – Taking a Patient's History (Part 2+3)

Nr.	Item	Supplementary Information
2.1	The physician greets the patient in a polite manner.	Greeting: salutation; non-verbal signals appropriately polite: uses his own name; the name of the patient<br>*Ex.: Hello Mr. Lauten, I am the attending physician and my name is [give name]. / Good morning / afternoon, my name is [give name]. You are Mr. Lauten, is that right?*
2.2	The physician addresses the patient in a respectful way.	The physician remains friendly and patient
2.3	The physician treats the patient as a mature and competent conversation partner.	The physician avoids lecturing the patient / isn't patronizing and gives the patient ample time to express him- / herself
2.4	The physician uses expressions that are in a language commonly understood by the patient (at least once the patient asks him / her to do so).	*Ex.: Loss of appetite* instead of *inappetence*; *painful swallowing* instead of *Odynophagia; Chickenpox* instead of *Varicella*
2.5	The physician gives verbal or non-verbal feedback cues.	Remarks such as „yes, yes", „sure"; nodding his /her head or the like
2.6	The physician ensures (through paraphrasing or summarizing) that he has correctly understood the information given by the patient.	Repeats information<br>*Ex.: Let me briefly summarize why you have come to us today: You have been feeling increasingly tired throughout the past week… / Swallowing has been difficult for you, am I correct?*<br>Summarizes questions<br>*Ex.: You want to know if…*
3.1	There is the overall impression that the medical condition of the patient has been captured sufficiently.	Irrespective of the linguistic interpersonal level and level of understanding, the act of communicating was successful regarding the use of questions to solicit information and the prompting of the patient to speak
3.2	All in all, the communicative behavior of the physician was appropriate and clear.	Irrespective of whether or not the use of questions to solicit information and the prompting of the patient to speak succeeded, the act of communicating was successful regarding the linguistic interpersonal level and the level of understanding

Comments:
